# Cascade reaction triggering and photothermal AuNPs@MIL MOFs doped intraocular lens for enhanced posterior capsular opacification prevention

**DOI:** 10.1186/s12951-023-01897-0

**Published:** 2023-04-24

**Authors:** Yueze Hong, Qiuna Fang, Ting Bai, Peiyi Zhao, Yuemei Han, Quankui Lin

**Affiliations:** National Engineering Research Center of Ophthalmology and Optometry, School of Biomedical Engineering, School of Ophthalmology and Optometry, Eye Hospital, Wenzhou Medical University, Wenzhou, 325027 China

**Keywords:** Posterior capsular opacity, Synergistic therapy, Intraocular lens, Metal–organic framework, Photothermal effects

## Abstract

Posterior capsular opacification (PCO) is the most common complication after cataract surgery. Present strategies can’t meet the clinical needs of long-term prevention. This research reports a novel intraocular lens (IOL) bulk material with high biocompatibility and synergistic therapy. Gold nanoparticles (AuNPs) doped MIL-101-NH_2_ metal–organic frameworks (MOFs) (AuNPs@MIL) was firstly fabricated via in situ reductions. Then the functionalized MOFs were uniformly mixed with glycidyl methacrylate (GMA) and 2-(2-ethoxyethoxy) ethyl acrylate (EA) to form the nanoparticle doped polymer (AuNPs@MIL-PGE), and which was used to fabricate IOL bulk materials. The materials’ optical and mechanical properties with different mass contents of nanoparticles are investigated. Such bulk functionalized IOL material could efficiently remove residual human lens epithelial cells (HLECs) in the capsular bag in the short term, and can prevent PCO on demand in the long run by near-infrared illumination (NIR) action. In vivo and in vitro experiments demonstrate the biosafety of the material. The AuNPs@MIL-PGE exhibits excellent photothermal effects, which could inhibit cell proliferation under NIR and doesn’t cause pathological effects on the surrounding tissues. Such functionalized IOL can not only avoid the side effects of the antiproliferative drugs but also realize the enhanced PCO prevention in clinical practice.

**Supplementary Information:**

The online version contains supplementary material available at 10.1186/s12951-023-01897-0.

## Introduction

Posterior capsular opacity (PCO) usually occurs months to years after cataract surgery, causing blurred vision and even blindness, affecting patients’ quality of life [1–3]. According to statistics, the incidence of PCO after cataract surgery in adults reaches 10% to 70%, while in children, it is as high as 100% [4]. Thus, considering the current mainstream Nd: YAG laser capsulotomy for PCO treatment [5], which might cause irreversible sequelae [6], how to prevent PCO effectively has become the research focus [7, 8]. The leading cause of PCO is the migration, proliferation, and epithelial-mesenchymal transition (EMT) of the human lens epithelial cells (HLECs) remaining in the capsular bag on the surface of the implanted intraocular lens (IOL) after surgical removal of the lens [9]. IOLs are ideal therapeutic platforms as intraocular implant materials in cataract surgery. Currently, most of the strategies for inhibiting PCO by IOL focus on surface coating and improving drug-loading methods [10–12]. Although other researchers have achieved remarkable results, certain drawbacks still cannot be ignored, namely long-term effectiveness [13]. This is mainly because a single treatment method is challenging to remove the residual HLECs in the postoperative capsular bag without causing side effects [14]. Hence, there is a more urgent need to enhance the inhibition of PCO development by synergistic therapy through multiple pathways while avoiding any possible side effects.

In ophthalmic disease treatment, non-invasive photothermal therapy (PTT) has broad clinical application prospects [15, 16]. Noble metal nanoparticles, especially gold nanoparticles (AuNPs), have been widely studied for PTT as a photothermal agent (PTA) due to their unique surface plasmon resonance (SPR) phenomenon [17] that enhances radioactive absorption and scattering properties [18–20]. In addition, AuNPs have been shown to obtain good glucose oxidase (GOD) mimics as well [21]. AuNPs oxidize glucose to gluconic acid through the exact catalytic mechanism as GOD, namely the two-step reaction of glucose dehydrogenation and subsequent reduction of O_2_ to H_2_O_2_ by two electrons [22]. Compared with the disadvantage that natural enzymes are quickly inactivated at high temperatures, AuNPs are undoubtedly excellent substitutes for GOD. However, under physiological conditions, its enzymatic activity will gradually lose due to solid protein adsorption, which also limits the application of AuNPs in clinical therapy [23].

In the past decades, crystalline materials with periodic network structures formed by self-assembly of metal ions and organic ligands based on coordination bonds [24], MOFs, have high porosity and low density, large specific surface area, and other advantages. It is widely used in gas storage, separation, catalysis, drug delivery, and sensing [25–27]. Nevertheless, MOFs are readily decomposed under peripheral stimuli (e.g., pH) [28]. Taking advantage of this unstable nature provides a new idea for constructing a new MOF therapeutic system.

**Scheme 1:**
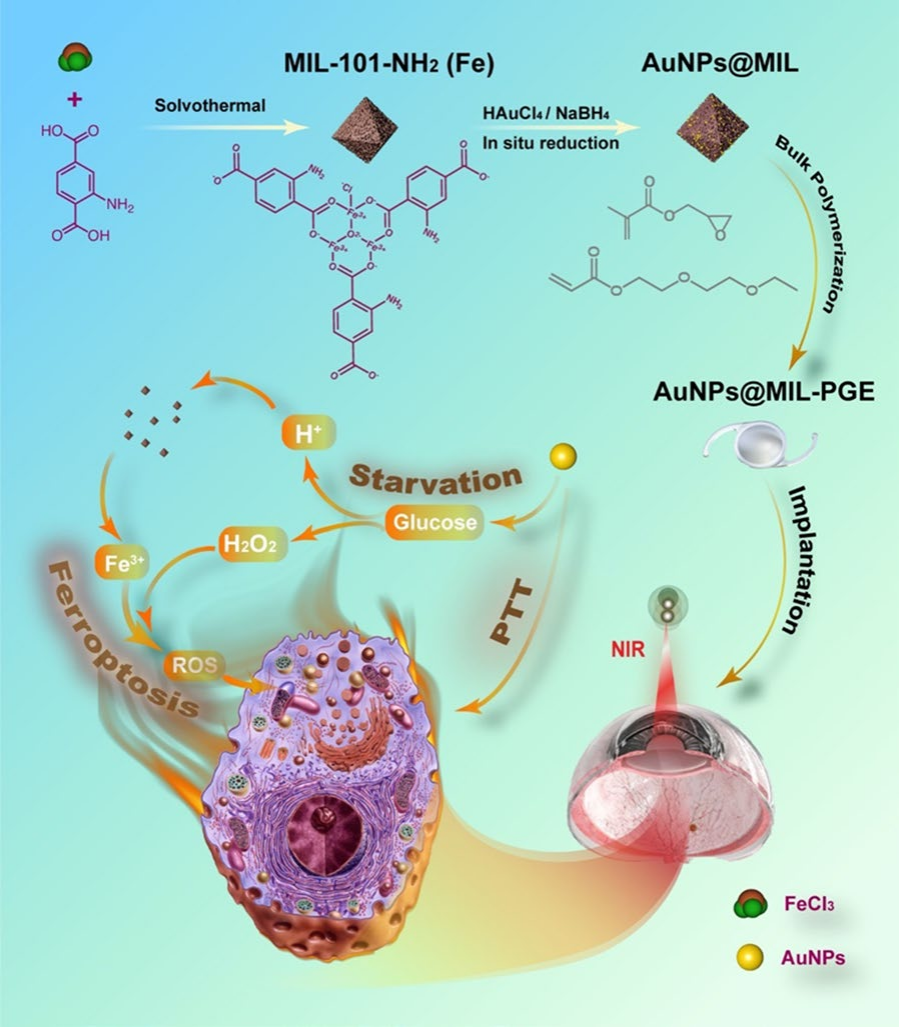
Schematic illustration of the cascade activation based on nanozyme-modified MOF(Fe) particles for synergistic starvation/ferroptosis/photothermal therapy via cascade reactions (1–3) in cells

Herein, a MOF-IOL material with multi-pathway therapeutic effects was designed to synergistically prevent PCO by starvation/ferroptosis/PTT (Scheme 1). Specifically, based on the characteristics of the internal channels of MIL-101-NH_2_ (MIL), AuNPs grow in MIL through in-situ reduction. This strategy can not only prevent the aggregation of nanoparticles but also effectively exert their glucose oxidase-like activity, consume glucose and oxygen in the bag, and competitively inhibits the proliferation of HLECs. It is generally believed that oxygen consumption will increase when cells proliferate, and cells will undergo anaerobic respiration in a relatively hypoxic environment to produce lactic acid, which acts together with gluconic acid to reduce the pH of the cell microenvironment further. Due to the instability of the coordination linkage in an acidic environment, MIL will gradually decompose to produce Fe^3+^, which goes through the Fenton reaction with H_2_O_2_ generated from the oxidation of glucose by AuNPs, and promotes lipid peroxidation (LPO) [29] in mitochondria. The mitochondrial morphology is destroyed, resulting in the cellular redox imbalance, causing programmed cell death, also known as ferroptosis [30], which further scavenges HLECs on the surface of the IOL. Synergistic therapy facilitated by a three-step cascade is given in Reactions 1–3. Furthermore, the photothermal effect of AuNPs is utilized to rapidly increase the temperature of the material to above 45℃ through the excitation of near-infrared light (NIR) at 808 nm, which promotes the apoptosis of the HLECs and achieves the purpose of artificially controlling the further development of PCO in a long-term time range.

1 $$Glucose+{O}_{2}+{H}_{2}O\stackrel{AuNPs}{\to }Gluconic\,acid\left({H}^{+}\right)+{H}_{2}{O}_{2}$$

2 $$AuNPs@MIL\stackrel{ {H}^{+}}{\to }{Fe}^{3+}+AuNPs+other\,products$$

3 $${H}_{2}{O}_{2} \stackrel{{Fe}^{3+}/{H}^{+}}{\to }ROS+{H}_{2}O$$

After introducing AuNPs@MIL into the poly (glycidyl methacrylate)-co-2-(2-ethoxyethoxy) ethyl acrylate (PGE) system, novel IOL bulk materials with synergistic therapeutic effects were prepared by radical polymerization. In our previous work [31, 32], the reliability of PGE as a novel IOL bulk material was verified. After AuNPs@MIL was doped into the IOL bulk material PGE, the MIL structure could not only prevent the inevitable agglomeration during the preparation of AuNPs but also prevent the deactivation caused by protein adsorption. And when MIL is used as a carrier platform to deliver AuNPs, it can also participate in the cascade reaction after its self-degradation and inhibit PCO through multiple pathways. In conclusion, the present research design takes full advantage of the unique properties of AuNPs to demonstrate the success of preventing PCO through cascade reactions and PTT in vivo/in vitro experiments. This strategy realizes the effective combination of short-term postoperative automatic prevention and long-term manual intervenability, which is expected to achieve effective prevention of PCO for an extended period after cataract surgery in the clinic.

## Materials and methods

### Materials

2-(2-Ethoxyethoxy) ethyl acrylate (EA), 2,2'-Azobis (isobutyronitrile) (AIBN), poly (ethylene glycol) diacrylate (PEGDA), sodium borohydride (NaBH4), and glycidyl methacrylate (GMA) were purchased from Sigma-Aldrich. N, N-Dimethyl-formamide (DMF), 2-aminoterephthalic acid (NH_2_-BDC), iron chloride hexahydrate (FeCl_3_· 6H_2_O), ascorbic acid,1,10-phenanthroline, and gold chloride trihydrate (HAuCl_4_ $\cdot$ 3H_2_O) were obtained from Aladdin. Commercial hydrophobic acrylate foldable IOLs were bought from Suzhou 66 Vision Tech Co., Ltd (66VT®, FV- 60A, the diameter of the optical region was 6 mm). Cell culture medium DMED/F12 (1: 1), fetal bovine serum (FBS), trypsin, penicillin, and streptomycin were acquired from Giboc. Cell counter kit-8 (CCK-8) and phosphate buffer saline (PBS) were bought from Invitrogen. Hoechst 33342 staining solution for live cells and mitochondrial membrane potential detection kit (JC-1) were obtained from Beyotime Biotechnology. All the eye drops used on animals after the operation were purchased from the Eye Hospital of Wenzhou Medical University.

### Preparation of nanoparticles

MIL can be synthesized by hydrothermal reaction. Briefly, 2.5 mmol FeCl_3_· 6H_2_O, 1.24 mmol NH_2_-BDC, and 15 mL DMF were first mixed and sonicated for 30 min. After the solution mixture was completely dissolved, it was put into a 30 mL Teflon-lined stainless-steel autoclave and reacted at 110 $\mathrm{^\circ{\rm C} }$ for 24 h. After the reaction, centrifuge at 12,000 rpm for 5 min to obtain a brown crude product. Centrifuged and washed twice with DMF and absolute ethanol and dried in a vacuum oven at 70 $\mathrm{^\circ{\rm C} }$ for 24 h to get MIL-101-NH_2_ (Fe). AuNPs@MIL was prepared according to the following methods. 200 μL of 10 mM HAuCl_4_ was added to 30 mL of 1 mg/mL MIL aqueous solution, stirred in an ice bath for 2 h, centrifuged, washed with ultrapure water, and then dispersed in 30 mL of water. Stir vigorously, add a freshly prepared 30 μL NaBH_4_ (0.1 M) aqueous solution, wash twice with ultrapure water, and finally centrifuge at 12,000 rpm for 5 min, and collect the product AuNPs@MIL after vacuum drying.

### Preparation of AuNPs@MIL-PGE

The copolymer AuNPs@MIL-PGE was obtained by radical polymerization with AIBN as the initiator. The steps are as follows: Mix the GMA and EA pretreated with the inhibitor remover at a ratio of 6:4, add the crosslinker agent PEGDA (10 wt%), the initiator AIBN (0.5 wt%), and AuNPs@MIL, ultrasonicate for 10 min to make a well-dispersed mixed solution. Continue stirring on a magnetic stirrer for 30 min. The N_2_ flow was continued for 30 min to remove O_2_ from the solution. The hybrid solution was then poured into a mold of two glass pieces separated by a 0.5 mm spacer, and polymerization was carried out at 60 $^\circ{\rm C}$ for 24 h. After that, the material was soaked in absolute ethanol to remove unreacted monomers and washed with ultrapure water. The AuNPs@MIL-PGE were dried in a vacuum oven to constant weight.

### Characterization

SEM images were photographed using a scanning electron microscope (FEI, QUANTA 650) with an accelerating voltage of 20 kV. TEM images and elemental analysis were recorded on an electron microscope (FEI, Talos F200X G2) operating at 100 kV. XPS and XRD patterns were acquired on an X-ray photoelectron spectrometer (Thermo Scientific, K-Alpha) and X-ray diffractometer (Panalytical, X'Pert PRO MPD). Zeta potential changes and particle size were detected o by Dynamic Light Scattering (Malvern, Zetasizer Lab). The optical transmittance was detected using a UV–Vis spectrophotometer (SHIMADAZU, UV-1780). The refractive index was seen using an Abbe refractometer (ATAGO, NAR-1T solids). Thermogravimetric analysis (TGA) was measured under a Nitrogen flow at a heating rate of 10 $\mathrm{^\circ{\rm C} }$/min up to 800 $\mathrm{^\circ{\rm C} }$ by the thermogravimetric analyzer (NETZSCH, STA 449F3). The glass transition temperature was detected by differential scanning calorimetry (TA, dsc250).

### Mechanical property measurement

After the AuNPs@MIL-PGE was made into a dumbbell shape with a thickness of 0.5 mm, the elongation and elastic modulus of each feeding ratio were tested on a universal testing machine, and the stress–strain curve was drawn. Shape memory capacity was assessed according to the bending test described in the literature [33]. AuNPs@MIL-PGE (50.0 × 10.0 × 0.5 mm) bends and maintains this shape at 37 $\mathrm{^\circ{\rm C} }$. The sample was then deformed and put into ice water for rapid cooling to relieve the deformation stress. Finally, the deformed sample was rapidly heated to the test temperature (37 $\mathrm{^\circ{\rm C} }$), and a timer measured the time from the deformed shape to its original form.

### Glucose oxidation assay

The glucose oxidation reaction was detected by the chronoamperometry method using an electrochemical workstation (CH Instruments). The procedure was as follows: the Platinum working Electrode, Ag/AgCl reference electrode and Platinum wire counter electrode were inserted into AuNPs@MIL solution (1 mg/mL, ultrapure water) together, and the current change was timed for 180 s. After that, 600 μL of glucose solution (10 mg/mL, ultrapure water) was added every 180 s, and the change of current response as a function of time was detected and recorded. Furthermore, the glucose oxidation effect after AuNPs@MIL doping into IOL materials was achieved by detecting pH changes in the solution. Glucose was added to 30 mL of ultrapure water at a concentration ratio of 0–10 mg/mL, and then five AuNPs@MIL-PGE (0.2 wt%, Φ = 6 mm) discs were added. After ten days of reaction at room temperature, the pH of the solution was detected using a pH meter.

### Detection of Fe^3+^ concentration

The steps to obtain the standard curve are as follows: 4 mM FeCl_3_ was diluted with ultrapure water to different concentrations, reduced with ascorbic acid (10 mM, 1 mL), and reacted with o-phenanthroline (0.1%, 1 mL) after 5 min of reaction. The maximum absorption peak was detected at 512 nm using a UV–Vis spectrometer, and the standard curve of Fe^3+^ concentration and absorbance was obtained. In order to detect the release of Fe^3+^, MIL-PGE (Φ = 6 mm) disks were immersed in 1 mL buffers with pH values of 7.4 and 4.5. At a predetermined time, 200 μL of the leaching solution was added to ascorbic acid to react for 5 min to reduce Fe^3+^ in the solution to Fe^2+^, and then the mixture was reacted with o-phenanthroline. The amount of Fe^3+^ released was calculated from the absorbance at 512 nm.

### Detection of reactive oxygen species (ROS) generation

ROS generation was detected by 9,10-Dimethylanthracene (DMA) fluoro-photometry. DMA was dissolved in DMF, prepared to 0.1 mg/mL, and stored in a refrigerator at 4 $\mathrm{^\circ{\rm C} }$. The DMA concentration was diluted to 0.05 μg/mL with glucose-containing PBS solution (1 mg/mL) for detection. AuNPs@MIL-PGE (Φ = 6 mm) was placed on the bottom of the fluorescence cuvette, and 2 mL of DMA was added. The fluorescence intensity at 432 nm was detected using a Fluorescence Spectrophotometer (HITACHI, F-4600) at the corresponding time interval, and a fluorescence intensity-time curve was drawn.

### Photothermal function and stability research

The thermal effect of the material was excited by irradiating AuNPs@MIL-PGE (Φ = 6 mm) with an 808 nm laser emitter at different powers for 60 s, and an infrared thermal imager (FOTRIC, 220 s) was used to capture and record the thermal imaging pictures at each time node in real-time. The following two methods verified stability. First, the AuNPs@MIL-PGE (Φ = 6 mm) was soaked in 40 mL phosphate buffer (pH = 7.4), and 10 mL of the leaching solution was taken for ICP-OES detection of gold in the solution at a preset time. And re-add a new equal volume of PBS. Second, the AuNPs@MIL-PGE was immersed in PBS (pH = 7.4) and taken out at a predetermined time. After drying the surface liquid, it was irradiated with 808 nm NIR (3.5 W/cm^2^) for 1 min, and the infrared image was observed and recorded by a thermal imager.

### In vitro cytotoxicity assay

The HLECs were cultured in a DMEM medium with 10% FBS and 1% penicillin–streptomycin at 37 $\mathrm{^\circ{\rm C} }$ in a humidified atmosphere of 5% CO_2_. The in vitro cytotoxicity of AuNPs@MIL was evaluated by the CCK-8 method. The HLECs were seeded into 96-well plates at a density of 5000 cells/well and incubated for 24 h at 37 $\mathrm{^\circ{\rm C} }$ in a CO_2_ incubator. The culture medium was then aspirated and washed once with PBS, after which 180 μL of DMEM with serum and 20 μL of AuNPs@MIL with different concentration gradients dispersed in PBS (pH = 7.4) was added to the wells and incubated at 37 $\mathrm{^\circ{\rm C} }$ for 24 h. Finally, the medium was blotted dry, and 100 μL of CCK-8 working solution was added to each well. After 2 h of incubation at 37 $\mathrm{^\circ{\rm C} }$, the absorbance at 450 nm was measured using a microplate reader to calculate the cell viability.

### In vitro detection of intracellular ROS production and changes in mitochondrial membrane potential

Cellular ROS production was induced using the extract and evaluated by a DCFH-DA staining kit. The leaching solution was prepared by soaking three sheets of AuNPs@MIL-PGE (Φ = 6 mm, 0.2 wt%) in 3 mL PBS (pH = 7.4) for 24 h. After the HLECs were seeded in 96-well plates at a density of 5000 cells/well and incubated for 24 h, 20 μL of leaching solution was added to each well, and the incubation was continued for 24 h. After that, the culture medium was removed, and DCFH-DA working solution was added and incubated for 30 min. After three washes with PBS, intracellular ROS generation was observed using an inverted fluorescence microscope (Leica, DMi8). Changes in mitochondrial membrane potential were detected by fluorescent probe JC-1. AuNPs@MIL-PGE (Φ = 6 mm) was plated at the bottom of a 96-well plate at a density of 2 × 10^4^ cells/well. After culturing at 37 $\mathrm{^\circ{\rm C} }$ for 24 h, the medium was aspirated and washed once with PBS. Add 200 μL of JC-1 staining solution and incubate at 37 $\mathrm{^\circ{\rm C} }$ for 20 min. Aspirate the liquid after incubation and wash twice with JC-1 staining buffer (1 ×). Finally, an inverted fluorescence microscope was used to observe the changes in membrane potential.

### Observation of cell staining after in vitro treatment

The ability of AuNPs@MIL-PGE to inhibit cell proliferation in vitro was evaluated by direct seeding and post-seeding overlay. The direct seeding method was to spread AuNPs@MIL-PGE (Φ = 6 mm) on the bottom of a 96-well plate, seed the plate at a density of 5000 cells/well, and culture at 37 $\mathrm{^\circ{\rm C} }$ for 24 h, blot dry the medium, add Hoechst33342 for staining. The working solution was incubated for 20 min and observed using an inverted fluorescence microscope. In the overlay method, after cells were seeded in a 96-well plate at a density of 5000 cells/well, the cells were cultured for 24 h until the cells were utterly adherent. Then the DMEM was aspirated, the AuNPs@MIL-PGE was lightly overlaid on the cells, and 200 μL of the medium was added. And the cells were incubated at 37 $\mathrm{^\circ{\rm C} }$ for 24 h. After that, the culture medium was removed, and Hoechst working solution was added to set for 20 min before observation using an inverted fluorescence microscope.

### Intraocular implantation experiments

The animal experiments were carried out under the approval of Animal Protection and Utilization Ethics Committee of Wenzhou Medical University. Ten New Zealand white rabbits weighing 2.5 ~ 3 kg were randomly divided into two groups. The phacoemulsification combined with IOL implantation was all performed by Dr. Han YM using a phacoemulsification instrument (Alcon, Laureate). Surgical instruments were sterilized by high-temperature steam, and all implant materials and sutures were sterilized by ethylene oxide. The surgical steps were the same as in our previous report [34, 35], and the polishing step was omitted to speed up the establishment of the PCO model. Preoperative use of proparacaine eye drops for ocular surface anesthesia and tropicamide eye drops for mydriasis. Postoperative use of compound tobramycin-dexamethasone and levofloxacin eye drops to prevent intraocular inflammation produced. The progress of PCO was observed at different times after operation using a slit lamp microscope. The functional integrity of the retina before and after surgery was assessed using electroretinography (ERG, RETI-Port21). In the 4th postoperative week, the rabbits were sacrificed under anesthesia, and the eyeballs were removed and stored at 4 $\mathrm{^\circ{\rm C} }$ in fixative. Subsequently, the eyeball was dissected to isolate the cornea, iris, retina, and lens capsular bag. The capsular bag was soaked in sucrose solution for 24 h and then frozen sectioned. Other eyeball tissues were dehydrated and then paraffined sectioned. All sections were stained with hematoxylin–eosin and photographed using a stereoscopic microscope (Nikon, SMZ18).

### Statistical analysis

All experiments were performed in triplicate, and experimental results were analyzed by GraphPad Prism 9. The statistical differences of different treatment groups were compared by ANOVA. *P < 0.05, **P < 0.01 and ***P < 0.001 were considered the differences were statistically significant.

## Results and discussion

### Synthesis and Characterization of Nanozyme-Modified MOF Materials

**Fig. 1:**
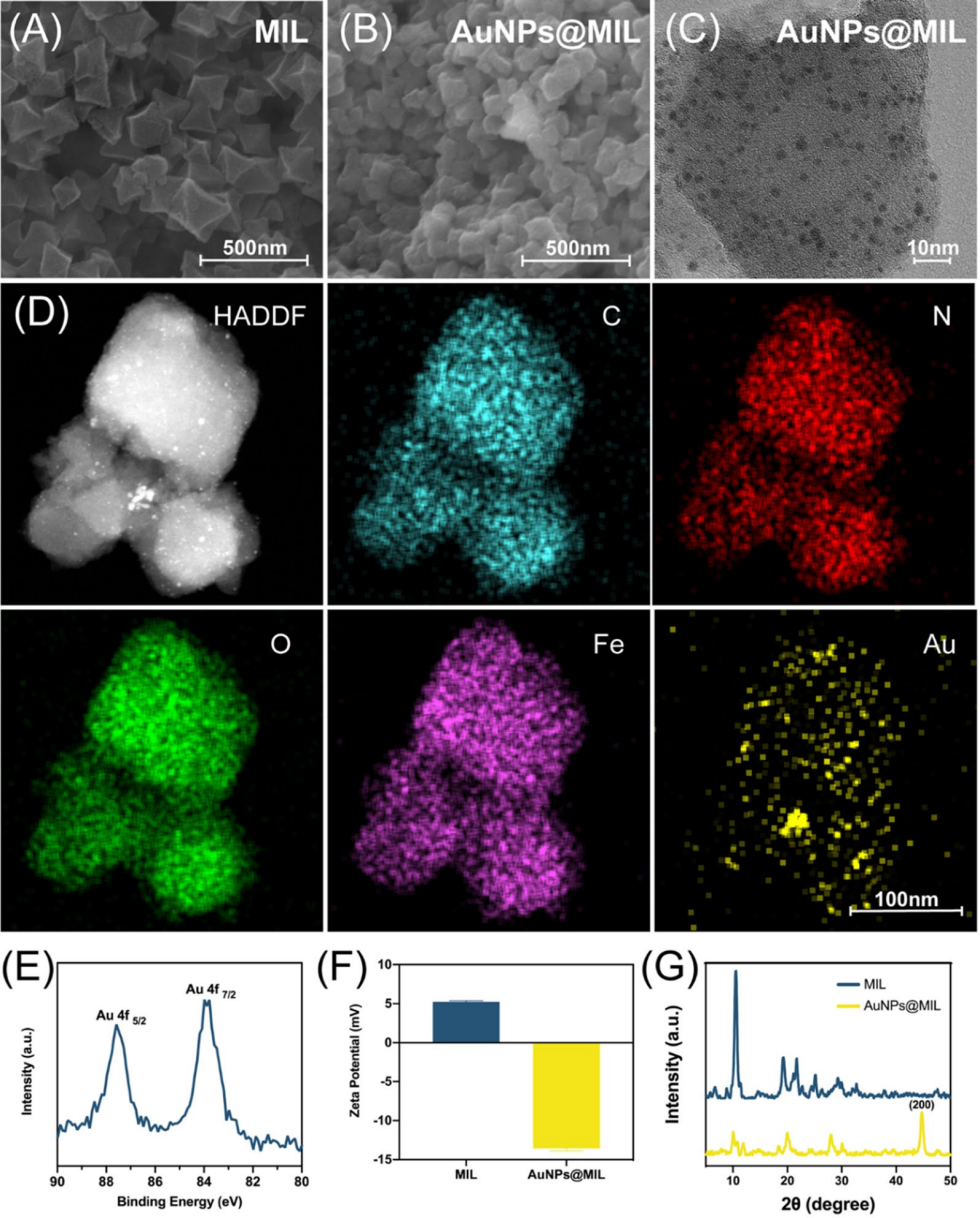
Materials characterization of nanoparticles. **A** SEM image of MIL. **B** SEM image of AuNPs@MIL. **C** TEM image of AuNPs@MIL. **D** The element mapping images of C, O, N, Fe, and Au. **E** The XPS spectra of AuNPs@MIL. **F** Zeta potential of MIL and AuNPs@MIL. **G** XRD patterns of MIL and AuNPs@MIL

In this work, MIL was synthesized according to the method described in the literature [36], and AuNPs@MIL was prepared by in situ reductions of NaBH_4_ [37]. The as-synthesized MIL could be observed under scanning electron microscopy (SEM) with a typical regular octahedral structure (Fig. 1A). Figure 1B indicated that after the in-situ reduction of HAuCl_4_, the particle size of the nanoparticles did not change significantly except that the surface became relatively rough. This was also evidenced by the particle size diagram (Additional file 1: Fig. S1). It should be noted that the large particle size of AuNPs@MIL may be related to particle aggregation. Transmission electron microscopy (TEM) images (Fig. 1C) proved that AuNPs were successfully prepared and loaded in MIL. The elemental mapping of AuNPs@MIL (Fig. 1D) demonstrated a uniform distribution of C, O, N, Fe, and Au elements. In addition, the Au (0) 4f_7/2_ peaks at 84.1 eV and the Au (0) 4f_5/2_ peaks at 87.9 eV in the XPS spectrum further revealed the successful in-situ reduction of Au^3+^ to zero-valent gold by NaBH_4_ (Fig. 1E). Surface zeta potential analysis also indicated that the successful loading of AuNPs on MIL (Fig. 1F), the negatively charged AuNPs reversed the surface charge of the initially positively charged MOFs. Eventually, by comparing the XRD patterns of MIL and AuNPs@MIL, it was clearly found that the characteristic peaks of MIL disappeared in the AuNPs@MIL pattern, and the 200-crystal plane diffraction peak attributed to Au appeared, which also verified that AuNPs were loaded onto the MIL surface in triumph. (Fig. 1G).

### Characterization and optimization of AuNPs@MIL-PGE

**Fig. 2:**
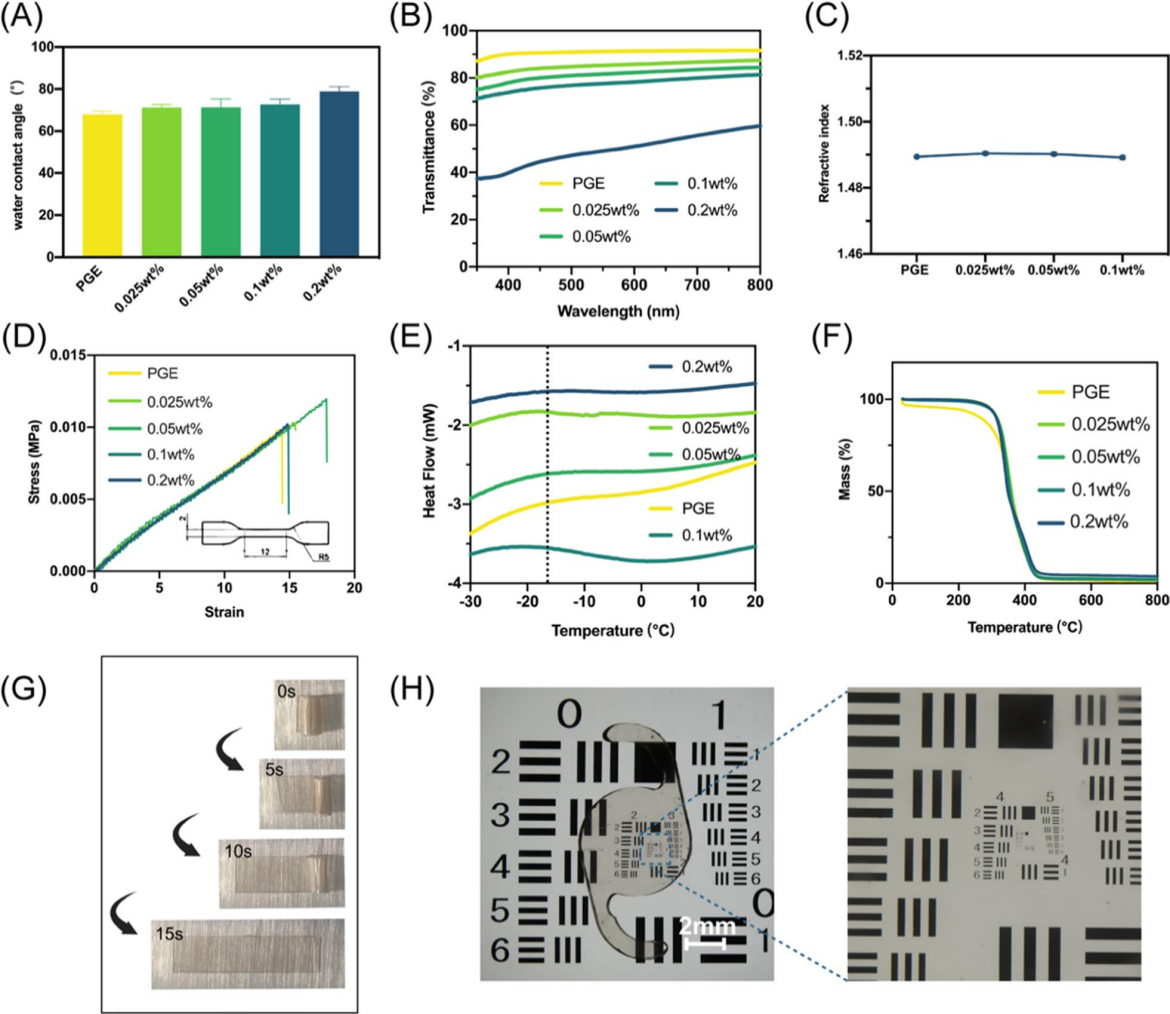
Physicochemical properties of IOL materials. **A** Water contact angle, **B** light transmittance and **C** refractive index of different mass contents of AuNPs@MIL-PGE. **D** Stress–strain curve of each groups of AuNPs@MIL-PGE and dumbbell-type cutting specifications for pull-up experiments (length 12 mm, width 2 mm). **E** Glass transition temperature, and **F** thermal decomposition curve of different mass contents of AuNPs@MIL-PGE. **G** The shape memory capability of AuNPs@MIL-PGE (0.1 wt%). **H** Image of the IOL placed on an optical resolution plate (objective magnification 0.75 $\times$) and the partial enlargement (objective magnification 4 $\times$)

To demonstrate that AuNPs@MIL-PGE can be implanted into the eye as an IOL material, a series of experiments were designed for material characterization. First, the surface hydrophilic and hydrophobic properties of IOLs affect the PCO process by simulating the adhesion of proteins and cells [10]. As shown in Fig. 2A, as the mass content of nanoparticles increased, the water contact angle (WCA) of the material surface increased from 67.94° ± 2.34° in the PGE group to 78.90° ± 2.18° in the 0.2 wt% group. These results indicate that the introduction of AuNPs@MIL increases the hydrophobicity of the material surface to a certain extent and allows for tighter attachment to the posterior capsule when implanted as an IOL, further restricting the growth and proliferation space of the HLECs. Furthermore, excellent optical properties remain necessary as an IOL material, which is crucial for adjusting the intensity and focus of light entering the eye through the IOL [38]. Optical transmittance (OT) and refractive index (RI) were used to evaluate the optical properties of the IOL (Fig. 2B, C). Since AuNPs@MIL is a tan opaque powder, the OT value further decreased with the increase of its mass content in PGE. However, at a lower concentration (< 0.1 wt%), the transmittance of more than 80% could still be maintained in the visible light wavelength range (390–780 nm). At the same time, the RI did not change significantly with the mass content and remained at around 1.49. Notably, the RI value of the 0.2 wt% group could not be obtained because its OT was too low.

Minor incision cataract surgery can effectively reduce the risk of postoperative intraocular inflammation and is an essential type of cataract surgery [39]. To meet the demands of minor incision surgery, the materials used to prepare IOLs must have excellent bending, folding, and shape recovery properties. The mechanical properties of the material were tested by tensile experiments, and the results manifest that the slope of the stress–strain curve of AuNPs@MIL-PGE was consistent with that of PGE (Fig. 2D), proving that the elastic modulus did not change significantly. The glass transition temperature (Tg) is the temperature at which polymer transitions from a glassy to a highly flexible state and can affect the further processability of the material [40]. The Tg of PGE did not change after incorporating AuNPs@MIL and remained at around – 18 ℃ (Fig. 2E). At the same time, thermogravimetric analysis (TGA) experiments proved the material has good thermal stability. The thermal decomposition temperature of each group is about 350 ℃ (Fig. 2F). The above experimental results imply that AuNPs@MIL-PGE is soft at both room temperature and human body temperature.

After a comprehensive evaluation, the increased content of AuNPs@MIL is beneficial to the effectiveness of the cascade reaction and does not affect other material properties without affecting the optical transmittance. Hence, a ratio of 0.1 wt% was chosen to continue the experiments. The mass of IOL made from one tablet of AuNPs@MIL-PGE is about 16.5 mg, of which the mass of AuNPs@MIL is about 16.5 µg according to the mass ratio conversion. The shape memory ability experiment demonstrated that the AuNPs@MIL-PGE could unfold and recover within 15 s after bending (Fig. 2G). The images taken after processing the material into IOL appearance and placing it on the resolution plate also intuitively show that AuNPs@MIL-PGE still has excellent transparency and satisfactory resolution compared to PGE (Fig. 2H & Additional file 1: Fig. S2). This suggested that the extraordinary bending ability and shape memory function of AuNPs@MIL-PGE not only satisfy the requirements of minor incision surgery but also could be speedily and gently unfolded after implantation in the eye, which can effectively shorten the operation time and will not cause excessive recovery speedily and damage the capsule. In conclusion, AuNPs@MIL-PGE can meet the basic requirements of IOL materials.

### Verify the occurrence of the cascade reaction

**Fig. 3:**
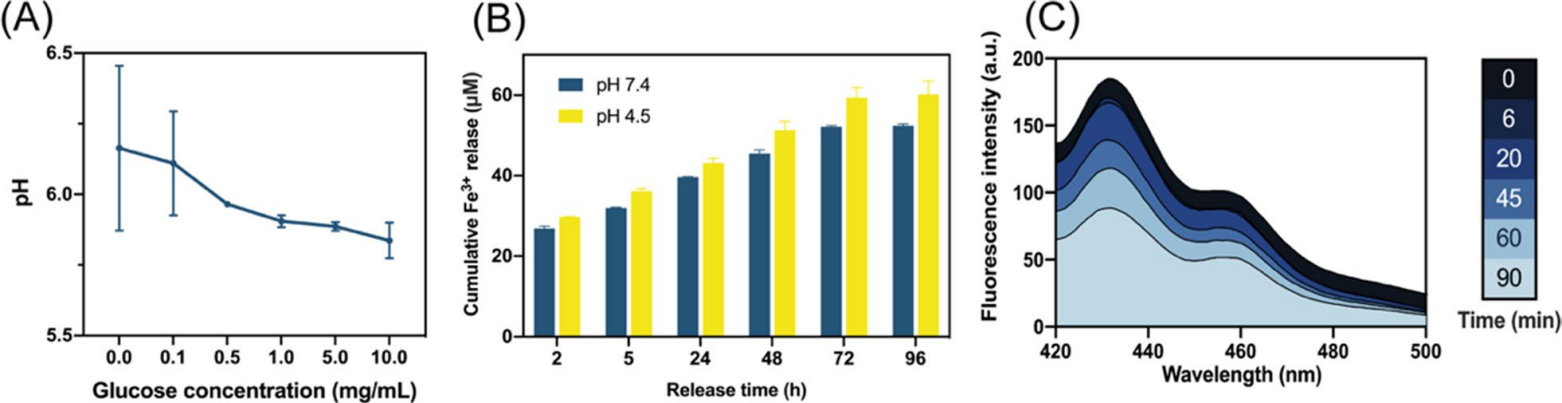
**A** pH values of AuNPs@MIL-PGE in Glu solutions with different concentrations. **B** Fe^3+^ degradation of MIL-PGE in different pH environments. **C** Changes in DMA fluorescence intensity after soaking in AuNPs@MIL-PGE for other times

To verify the occurrence of the designed cascade reaction, we detected the leading products in reactions (1–3), including the generation of gluconic acid (H^+^), Fe^3+^, and ROS. As expected, it is manifested in Fig. 3A, when AuNPs@MIL-PGE was immersed in a glucose solution with a concentration gradient of 0.1 to 10 mg/mL for a while, the pH in the solution increased with the glucose concentration, demonstrating the response (1) the formation of gluconic acid. The gold nanoparticle-catalyzed glucose oxidation reaction was also demonstrated by electrochemical methods (Additional file 1: Fig. S3). After each addition of a quantitative glucose solution to the AuNPs@MIL solution, a regular change in the current near the working electrode occurred. This is due to the hydrolysis of gluconic acid produced by the oxidation of glucose, resulting in fluctuations in the current response, while the current changes leveled off after the consumption of glucose. In contrast, when glucose solution was added to the group with ultrapure water as the control, no significant fluctuation in the current near the working electrode occurred because no oxidation reaction occurred.

In addition, since the coordination bonds that maintain the stability of nanoparticles are easily broken in an acidic environment, either the acidic microenvironment generated by cell proliferation or the gluconic acid caused by the oxidation of glucose by gold nanozymes will promote the degradation of nanoparticles and release the Fe^3+^. Using o-phenanthroline as a detector, Fe^3+^ released from the degradation of nanoparticles due to pH changes was investigated. The results are shown in Fig. 3B, and it can be seen that the acidic environment does promote Fe^3+^ release because the release amount at pH = 4.5 was always higher than that at pH = 7.4. To verify the occurrence of reaction (3), ROS detection was performed by fluorophotometry. DMA is a fluorescent compound (λex/λem = 395/432 nm), which can selectively react with ROS to generate a non-fluorescent endoxide, so the generation of ROS can be reflected by observing the change of fluorescence intensity at 432 nm [41]. It is also worth noting that since ROS is the final product of the cascade reaction, the generation of ROS also further proves the successful progress of reactions (1, 2). As can be seen from Fig. 3C, the cascade reaction of AuNPs@MIL-PGE successfully proceeded after soaking in a glucose solution, and the generated ROS accumulated with increasing soaking time.

### Photothermal effect and stability research

**Fig. 4:**
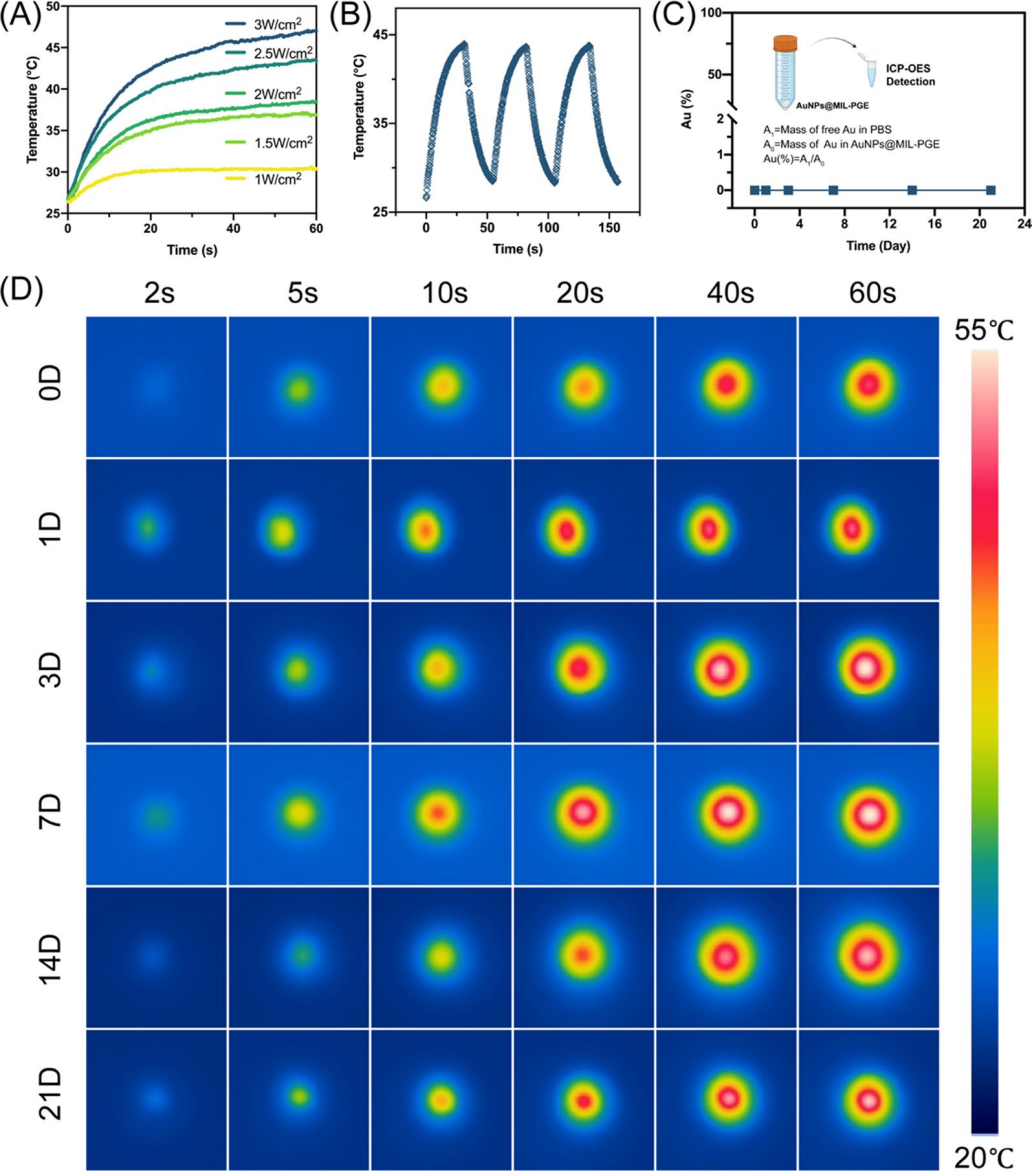
Photothermal effect and stability test of AuNPs@MIL-PGE. **A** The temperature curve of AuNPs@MIL-PGE under different illumination power. **B** The temperature-dependent curves of AuNPs@MIL-PGE were measured for three cycles under NIR irradiation (2.5 W/cm^2^). **C** ICP-OES measurement of the detachment of AuNPs from the IOL material. AuNPs@MIL-PGE was soaked in PBS, and the leaching solution was taken at different intervals to measure the Au content by ICP-OES. **D** Photothermal images of AuNPs@MIL-PGE after immersion in PBS for other times under 808 nm NIR irradiation (3.5 W/cm^2^)

To verify the photothermal effect of AuNPs@MIL-PGE, the material was fabricated into a 6 mm diameter disk, and the material was irradiated with 808 nm NIR. According to Fig. 4A, the AuNPs inside the material played the PTA role with the increased light power, making the material heat up rapidly. The temperature of AuNPs@MIL-PGE showed an increasing trend. Subsequently, the thermal stability of AuNPs@MIL-PGE and the optical functional damage caused by the photothermal effect was further investigated by three consecutive heating–cooling cycles. Specifically, NIR (2.5 W/cm^2^) irradiation for 30 s and cooling for 20 s. There was no significant difference in the temperature change for each cycle (Fig. 4B), indicating that the material has high thermal stability and that frequent light exposure does not cause functional damage to the material. The presence of gold was also not detected from the leachate by an inductively coupled plasma optical emission spectrometer (ICP-OES) (Fig. 4C). This indicates that AuNPs@MIL-PGE are highly stable, and the internal AuNPs will not be released from PGE with the decomposition of MOFs, showing good biosafety. As an IOL material, stability is critical for long-term functioning after implantation in the eye. The aqueous humor environment was simulated by soaking the AuNPs@MIL-PGE in PBS, which was taken out and irradiated with NIR at different times. The results show that AuNPs@MIL-PGE still has excellent photothermal performance after 21 days. As shown in Fig. 4D, as the irradiation time increased, the irradiation center region appeared pale yellow (31.6 $^\circ{\rm C}$) at the 2nd sec, red at the 20th sec (49 $^\circ{\rm C}$), and finally white at the 60th sec (52 $^\circ{\rm C}$). At the same time, due to thermal conduction, the temperature of the area around the wafer finally increased to 37.5 $^\circ{\rm C}$, which is close to the temperature of the human body. These results show that AuNPs@MIL-PGE has an excellent regional thermal effect. The heating region is confined to the illumination range and does not cause thermal damage to the surrounding tissues of the eye.

### In vitro cell experiments

**Fig. 5.**
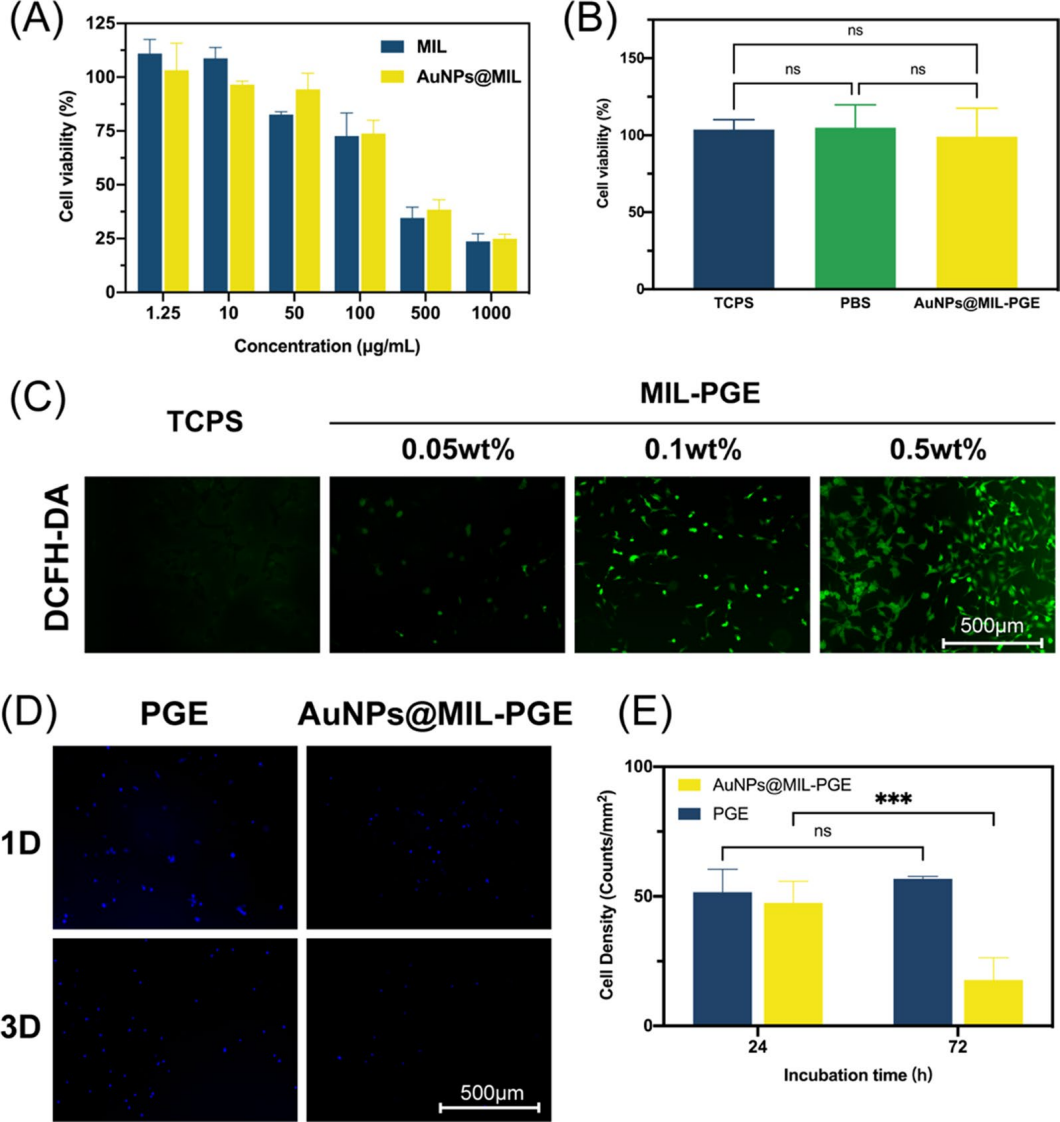
**A** Cytotoxicity of MIL and AuNPs@MIL at different concentrations. **B** Cell viability after being cultured with extracts of different materials. **C** DCFH-DA staining images of cells treated with MIL-PGE extracts with other contents. **D** Hoechst staining images of cells seeded on surfaces of different materials and cultured for a specific time. **E** Statistical analysis of cell counts after cells were seeded on surfaces of different materials and cultured for a particular period.

To evaluate the biosafety of nanoparticles, the effect of AuNPs on the viability of the HLECs at different concentrations was tested by the CCK-8 method. The results are illustrated in Fig. 5A, the cytotoxicity of AuNPs increased with increasing engagement, and at low concentrations, the nanoparticles exhibited high biosafety. Biocompatibility is an essential property of IOL materials, and the cell viability was calculated by the CCK-8 method after treatment with the material leaching solution. The leaching solution was obtained by soaking one AuNPs@MIL-PGE disk (0.1 wt%, Φ = 6 mm) in 5 mL PBS (pH = 7.4) for 24 h. The HLECs were inoculated into 96-well plates at a density of 5000 cells/well and incubated for 24 h. Then 20 $\mu$ L of the extract was added to culture for 24 h, 10% concentration of CCK-8 working fluid was added and incubated for 2 h, and then the OD value was measured by a microplate reader to calculate cell viability. Compared with the control group TCPS, there was no significant difference among the groups, proving that the cell viability was hardly affected by the leaching solution of AuNPs@MIL-PGE (Fig. 5B). Intracellular ROS accumulation is a significant feature of cells undergoing ferroptosis [28]. The intracellular ROS generation after treatment with different contents of MIL-PGE extract was evaluated by the DCFH-DA probe. As shown in Fig. 5C, as the range of MIL in PGE increased, the green fluorescence in cells increased, which was caused by the oxidation of non-fluorescent DCFH into cells by ROS to generate DCF with green fluorescence. At the same time, this phenomenon also proved that the increase of MIL content increases the dissociated Fe^3+^, which leads to the rise of intracellular ROS accumulation and further promotes the process of ferroptosis. The Hoechst staining further evaluated the ability of PGE to inhibit cell proliferation before and after loading AuNPs@MIL. The number of cells in the PGE group did not change significantly with the increase in culture time, which may be related to the hydrophobicity of the PGE material and the ability to inhibit cell migration (Fig. 5D, E). While the AuNPs@MIL-PGE group was cultured for another 72 h, the number of cells was significantly reduced compared with 24 h and considerably less than the PGE group. This proved that the AuNPs@MIL-PGE material could effectively remove the residual HLECs on the surface while retaining the excellent ability of PGE, whose good functionality of anti-adhesion and anti-cell migration ability has been demonstrated and introduced in detail in our previous work [32].

**Fig. 6:**
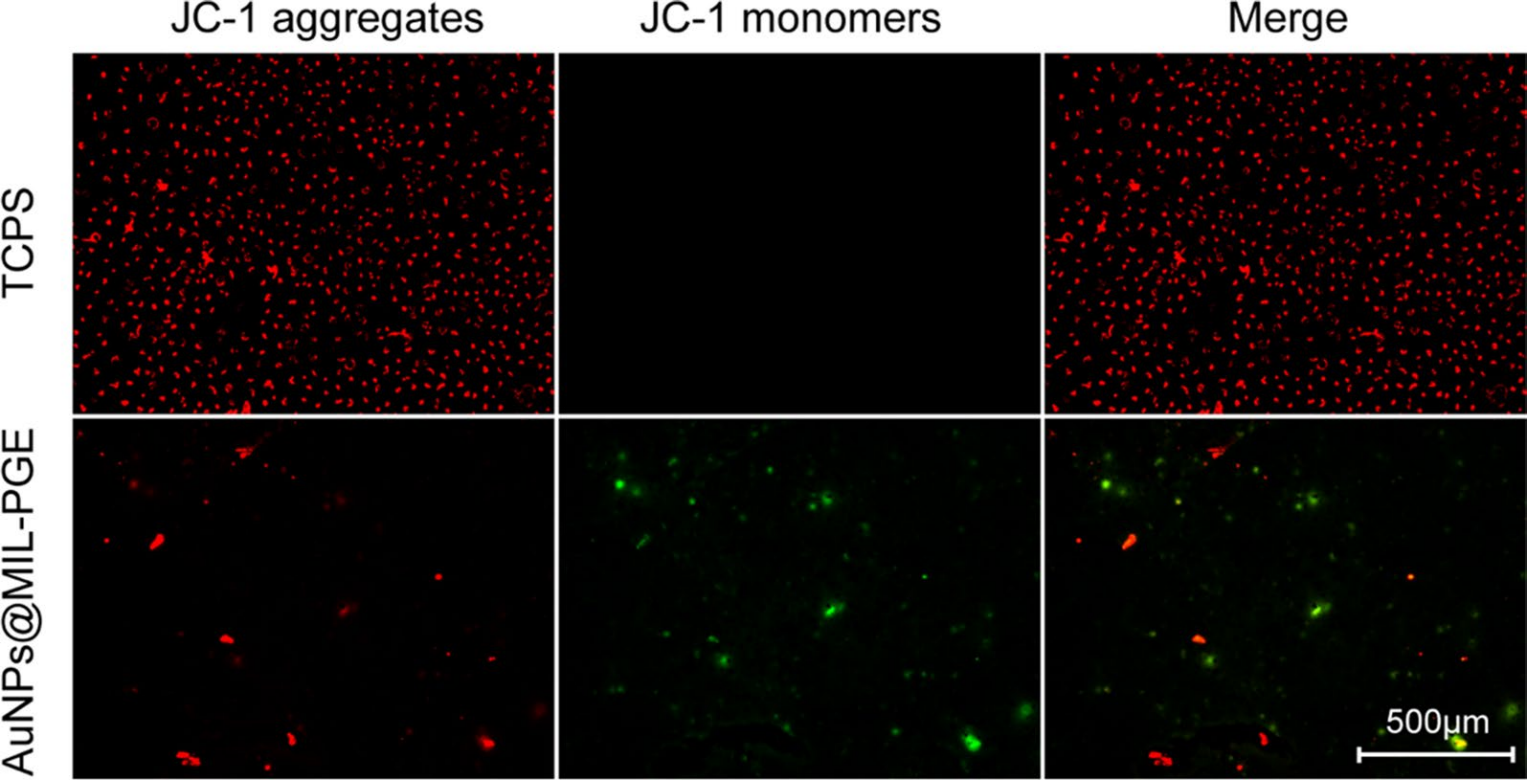
Fluorescence images detected by JC-1 probe after culturing HLECs on different materials for 24 h

The morphological change of mitochondria is one of the crucial features of ferroptosis in cells [42]. Lipid peroxidation caused by the accumulation of ROS will gradually destroy the standard shape of mitochondria. The change in the fluorescence color of the probe JC-1 can directly show the transformation of mitochondrial membrane potential. As depicted in Fig. 6, the cells in the TCPS group grew normally, the mitochondria maintained a high membrane potential, and JC-1 formed a polymer aggregated in the mitochondrial matrix, showing red fluorescence. On the other hand, cells cultured on AuNPS@MIL-PGE showed green fluorescence. This is because JC-1 cannot aggregate in the mitochondrial matrix but produce green fluorescence in its monomeric form. Taking the above results together, it can be proved that AuNPS@MIL-PGE triggers ferroptosis, leading to mitochondrial morphology changes and significantly reducing the mitochondrial membrane potential.

**Fig. 7:**
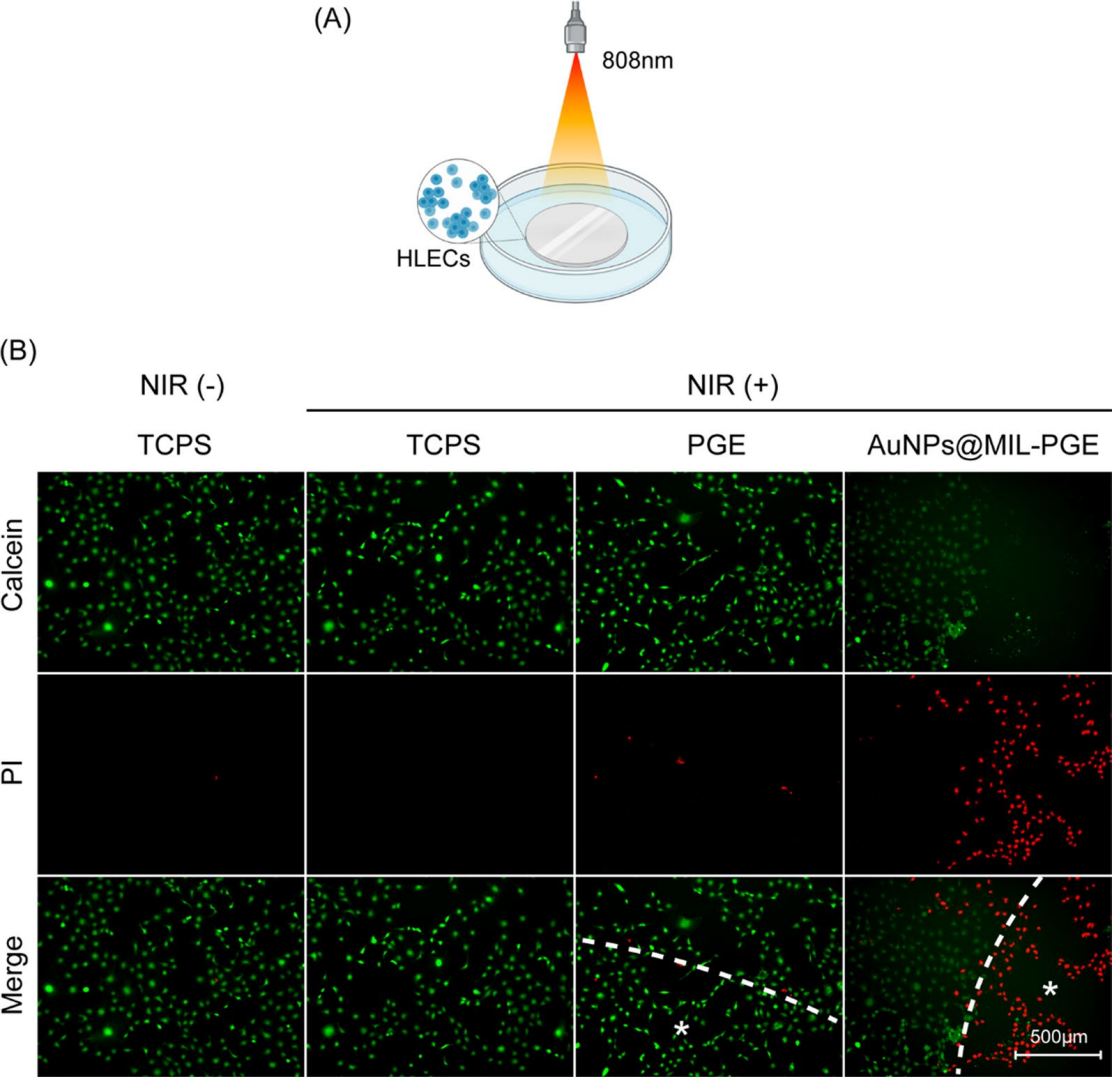
**A** Schematic illustration of the killing of the HLECs by AuNPs@MIL-PGE after light irradiation in vitro. **B** Live/dead staining images of LECs on the surface of PGE, AuNPs@MIL-PGE, and TCPS after different treatments (laser on & off). The symbol * indicates the material coverage area (808 nm, 3.5 W/cm^2^, 10 min).

The spatially controllable photothermal properties of AuNPs@MIL-PGE were evaluated by cell death/live staining after NIR irradiation. The HLECs were seeded at 2 $\times$ 10^5^ cells/well in 48-well plates and incubated for 24 h. AuNPs@MIL-PGE disks were placed on top of the cells and irradiated with 808 nm NIR (3.5 W/cm^2^) for 10 min (Fig. 7A). Calcein/PI staining was then performed and incubated in a CO_2_ incubator for 20 min. Observed and photographed by an inverted aberration microscope, the results show that the cells covered by AuNPs@MIL-PGE show red fluorescence due to death, and cells far away from AuNPs@MIL-PGE still have green fluorescence. In the control group, including the TCPS group and PGE group with NIR ( ±), the cells in the well plate were all alive (green signal), indicating that the function of killing the cells came from the light of AuNPs@MIL-PGE Thermal effects, rather than NIR irradiation, further demonstrate that NIR light is safe (Fig. 7B).

### In vivo animal experiment

**Fig. 8:**
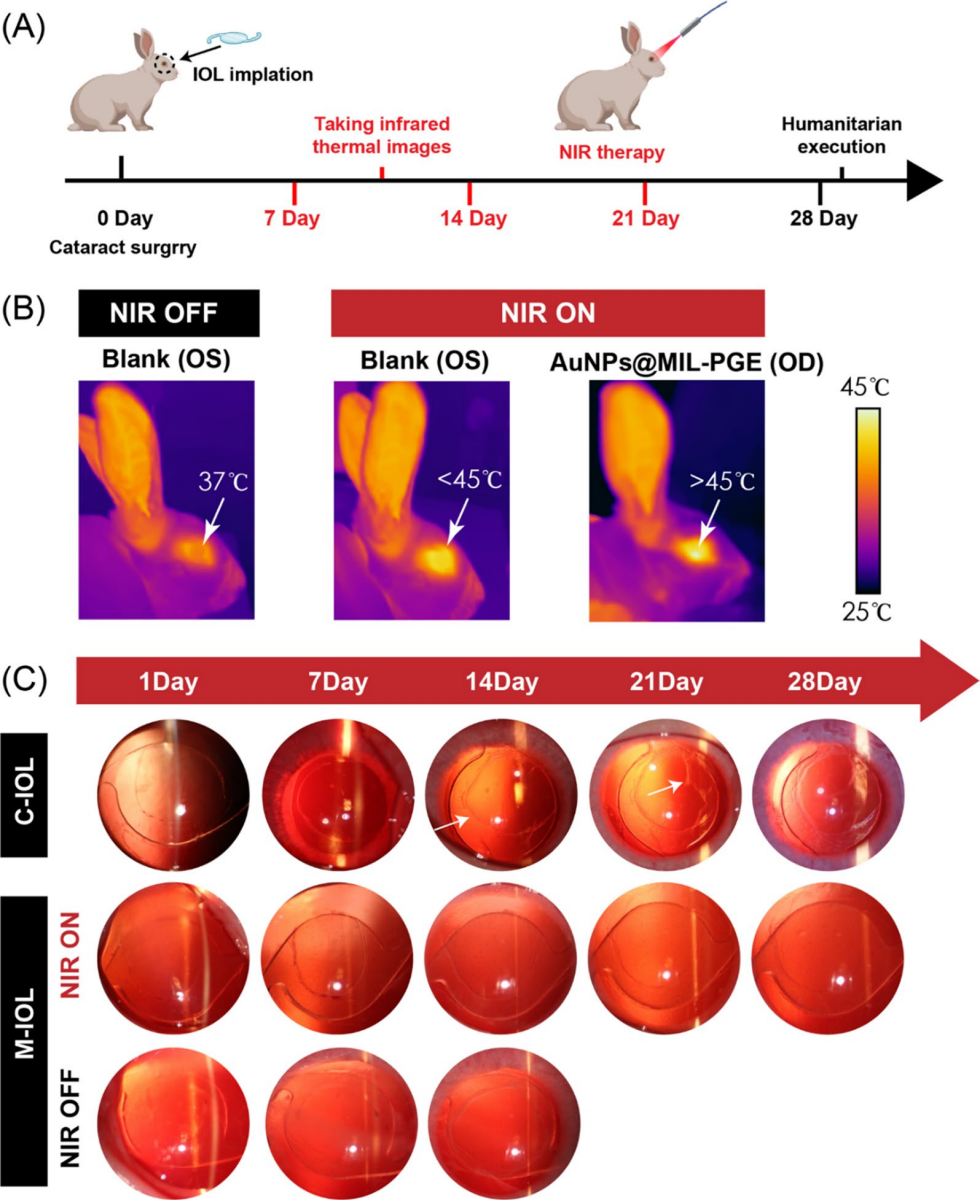
In vivo photothermal therapy for PCO prevention using IOL fabricated from AuNPs@MIL-PGE materials. **A** Timeline of cataract surgery and photothermal treatment in rabbits. **B** The infrared imaging pictures of rabbit eyes in different treatment groups. **C** Slit lamp microscope images of the OD of each group at different periods after surgery

Following the method reported in previous work, phacoemulsification and IOL implantation were performed in the right eye of New Zealand white rabbits to evaluate further the inhibition of PCO occurrence after implantation of IOLs prepared by AuNPs@MIL-PGE effect. Rabbits weighing 2.5 ~ 3 kg were randomly divided into C-IOL and M-IOL groups, with commercial IOL implanted in C-IOL group and AuNPs@MIL-PGE implanted in M-IOL group. On the 7th, 10th, 14th, and 21st days after the operation, the rabbits in the M-IOL group received NIR irradiation (808 nm, 3.5 W/cm^2^, 5 min). It was observed by a slit lamp microscope on the 7th, 14th, 21st, and 28th days after the operation, respectively (Fig. 8A). Additionally, due to the difference between the intraocular environment and the in vitro simulation, in order to more intuitively represent the heating performance of AuNPs@MIL-PGE under NIR illumination after implantation, an infrared imager was used to evaluate the temperature changes under different conditions. The results are shown in Fig. 8B. Under normal conditions, the temperature of the rabbit eye was around 37 $\mathrm{^\circ{\rm C} }$, and the temperature of the lens part was lower than that of the periphery. Due to the thermal effect of infrared light, the temperature around the normal ocular surface rose slightly, but still lower than 45 $\mathrm{^\circ{\rm C} }$ and would not affect cells. In the M-IOL group, the intraocular temperature rose to above 45 $\mathrm{^\circ{\rm C} }$ after 5 min of NIR irradiation, which showed the ability to inhibit cell proliferation [43]. To more intuitively assess the occurrence of postoperative PCO, the lens surface of the IOL at different periods was observed using a slit lamp microscope. Within one week after the operation, some rabbits developed corneal edema to varying degrees, but all of them resolved spontaneously a few days later. On the 14th postoperative day, the capsule in the C-IOL group was folded, and fibrous tissue adhesions were formed around the capsulorhexis, gradually increasing with the observation time (Fig. 8C). These above phenomena showed the rapid formation and progression of PCO, while the posterior capsule of the M-IOL group remained relatively transparent, and no apparent folds and fibrous tissue were observed within 28 days after surgery.

**Fig. 9:**
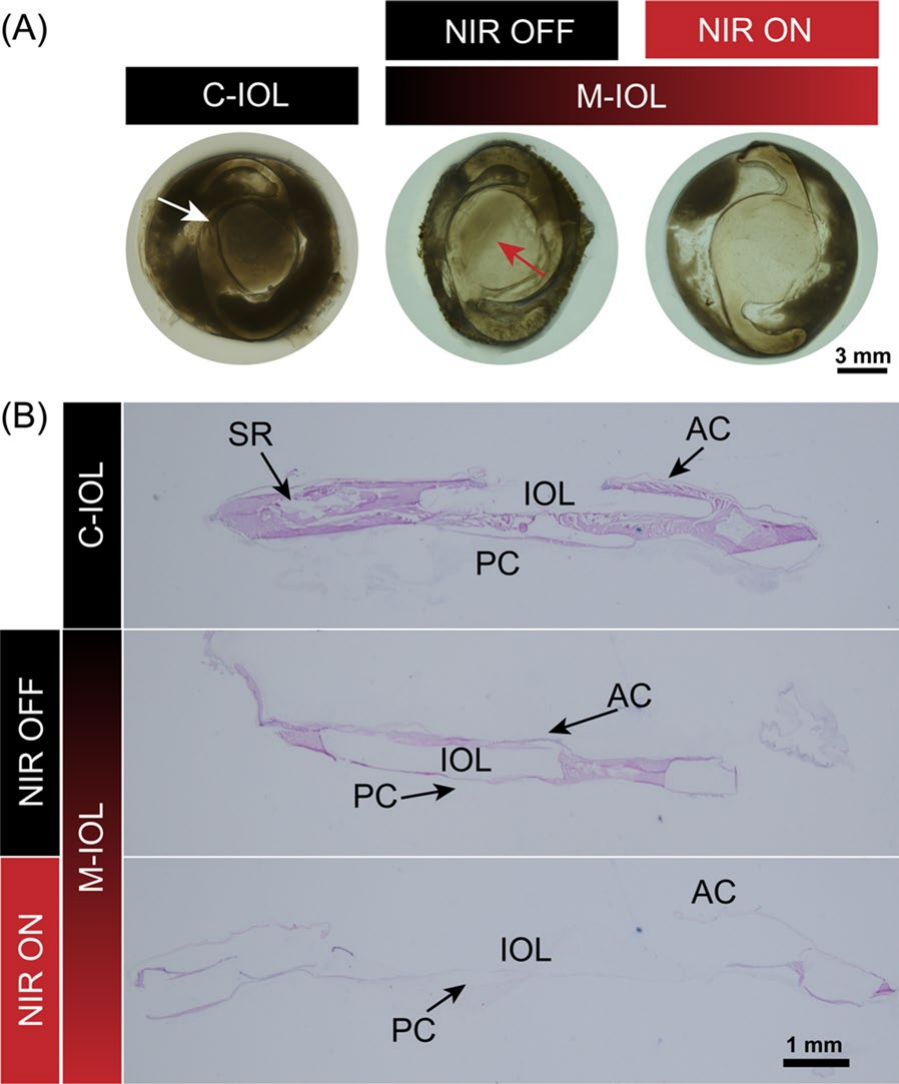
Evaluation of the ability to inhibit PCO after IOL implantation in each group. **A** Representative stereomicroscopic images of the lens capsular bag. **B** Representative images of H&E staining of the capsular bag after cryosection (*SR* Soemmering ring, *AC* anterior capsules, *PC* posterior capsules)

After the rabbits were euthanized, the eyeballs were removed. The capsular bag tissue was dissected anatomically, and the degree of PCO progression was observed under a stereomicroscope. As displayed in Fig. 9A, consistent with the slit-lamp observations, the HLECs in the C-IOL group proliferated and migrated, resulting in the occlusion of the IOL optical area by fibrotic tissue (marked by white arrows). Meanwhile, the optical center of the M-IOL group remained transparent whether NIR irradiation was performed. Notably, to observe the therapeutic effect of the cascade reaction in the short term, the NIR OFF group was sacrificed on the 20th day after the operation, and a little cortical hyperplasia began to appear at the point marked by the red arrow. As for the NIR ON group, no PCO progression was observed in the IOL surface area after a longer experimental time. Subsequently, these tissues were processed for H&E staining after cryosectioning. The results are demonstrated in Fig. 9B, and there was a large number of cortical hyperplasia on the surface of the commercialized IOL, which severely impeded the optical area and affected the visual quality. In the M-IOL group, proliferating cells in the pouch were significantly reduced when NIR irradiation was not used, manifesting the effectiveness of the cascade in inhibiting cell proliferation in the short term. Yet, after NIR irradiation, no HLEC proliferation was observed on the anterior and posterior capsules, and the optical center of the IOL was not obstructed in any way. To sum up, it is not difficult to find through the comparative analysis of each group that the synergistic cascade reaction of photothermal therapy can further enhance the preventive effect of PCO and more effectively inhibit the occurrence of PCO in a more extended time range.

**Fig. 10:**
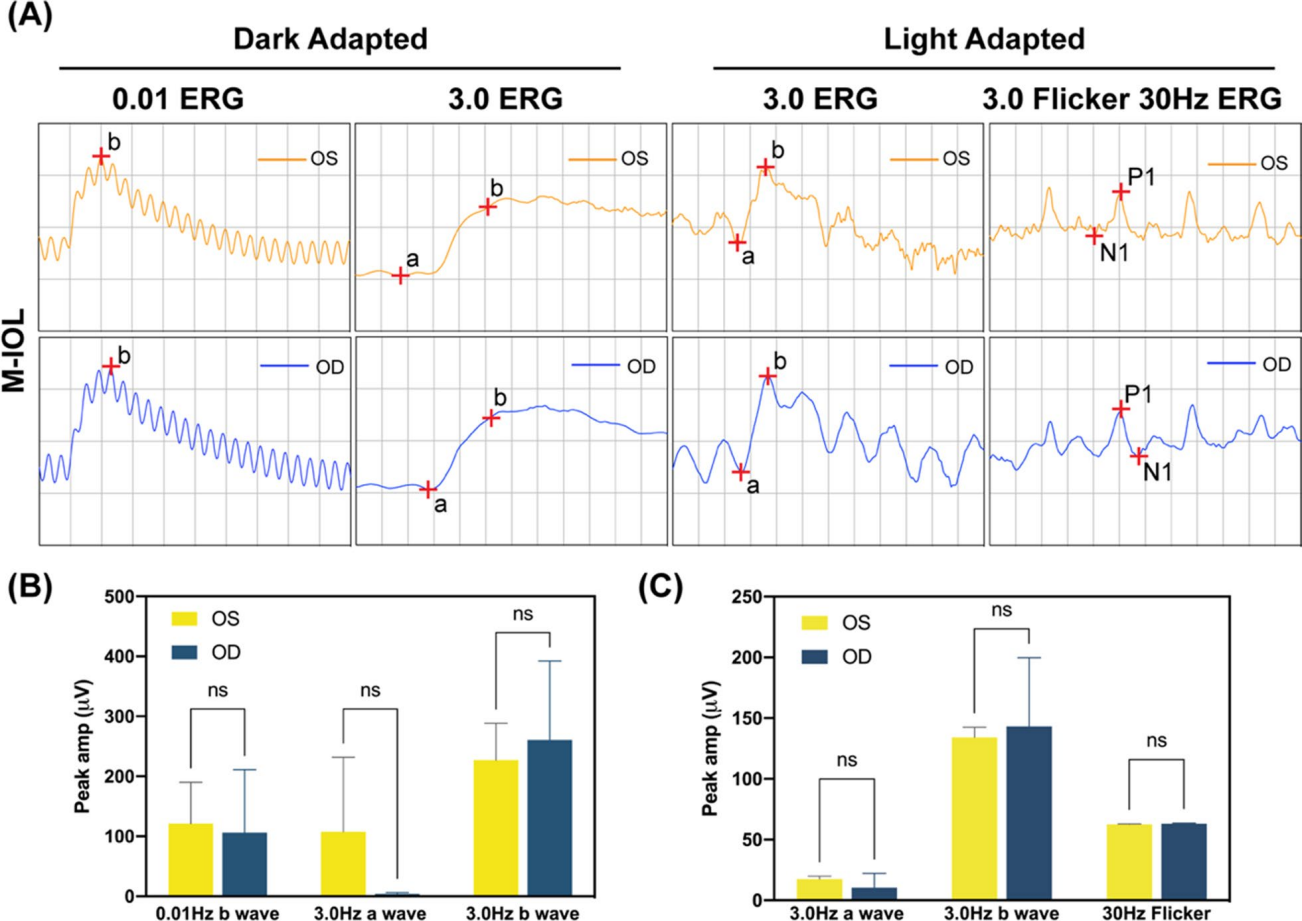
**A** Electroretinogram of the operated eye (OD) and normal eye (OS) in the M-IOL group. The peak changes of ERG at each stage after **B** dark adaptation and **C** light adaptation in the M-IOL group operated eyes and normal eyes

During the experiment, since the eye was directly irradiated with NIR light, and the nanoparticle degradation would produce small molecular species distribution in the eye as well, the actual safety of this strategy should be rigorously evaluated. Electroretinogram is a vital experiment to reflect the integrity of retinal function. Electroretinograms of the operated eye (OD) and non-operated eye (OS) were compared on postoperative day 30. As exhibited in Fig. 10A, the ERG amplitude and waveform of the OD in the M-IOL group were basically the same as those of the OS. After statistical analysis (Fig. 10B, C), it can be seen that there is no significant difference in the peaks detected at each stage between OD and OS after ERG undergoes dark adaptation and light adaptation. This indicates that after M-IOL implantation and NIR treatment, the retina of the operated eye can still respond to the light signal stimulation as normal eye, which proves that the material and treatment method are safe and reliable.

**Fig. 11:**
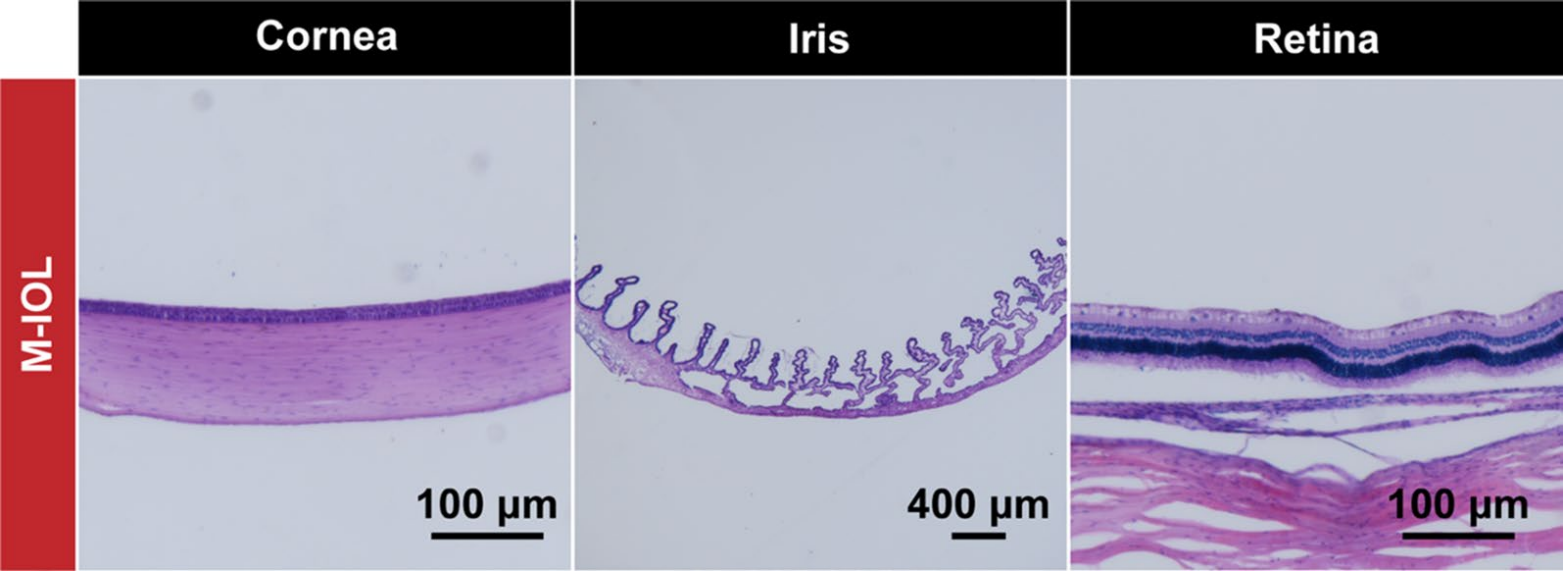
Representative H&E staining images of various eyeball parts after paraffin sectioning

Besides, the staining results of the paraffin-embedded eyeballed sections also revealed that the tissues (including the cornea, iris, and retina) remained morphologically intact at the cellular level, and no pathological changes occurred (Fig. 11). These results all proved that the application of M-IOL has satisfactory biosafety. The degradation of MIL and direct exposure to NIR light did not cause damage to retinal function or normal corneal morphology, nor did it affect the morphology and function of the tissues surrounding the eye.

## Conclusion

In summary, we propose a novel IOL material doped with nanoparticles, which can achieve high efficiency and long-term prevention of PCO through multi-channel combined therapy. AuNPs@MIL-PGE retains the good mechanical and physicochemical properties of PGE while taking advantage of the nanozyme and photothermal properties of AuNPs. It can remove residual HLECs in the capsular based on starvation/ferroptosis/photothermal therapy, which helps prevent PCO from being the root cause. In vitro cell experiments and animal intraocular experiments demonstrated that AuNPs@MIL-PGE had excellent cell scavenging ability and good biosafety. Histopathological sections also showed that the IOL bulk material did not affect the surrounding parts of the eye. This method of doping nanoparticles into IOL bulk materials provides a new idea for preventing PCO, which is very promising for biomedical applications.

## Supplementary Information

**Additional file 1: Figure S1.** Particle size diagram of MIL and AuNPs@MIL measured by DLS. **Figure S2. **Image of the IOL made from PGE placed on an optical resolution plate (objective magnification 0.75$\times$). **Figure S3. **The amperometric I-t curve of AuNPs@MIL (1 mg/mL) and H_2_O in presence of continuous addition of 10 mg/mL glucose solution at certain time intervals.

## References

[1] Konopińska J, Mynarczyk M, Dmuchowska DA, Obuchowska I (2021). Posterior capsule opacification: a review of experimental studies. J Clin Med.

[2] Wormstone IM, Wormstone YM, Smith AJO, Eldred JA (2020). Posterior capsule opacification: What's in the bag?. Prog Retin Eye Res.

[3] Toffoletto N, Saramago B, Serro AP (2020). Therapeutic ophthalmic lenses: a review. Pharmaceutics.

[4] Kisa A, Kisa S, Collaborators BVI, Samy A (2020). Trends in prevalence of blindness and distance and near vision impairment over 30 years: an analysis for the Global Burden of Disease Study. Lancet Glob Health.

[5] Ursell PG (2018). Three-year incidence of Nd:YAG capsulotomy and posterior capsule opacification and its relationship to monofocal acrylic IOL biomaterial: a UK Real World Evidence study. Eye.

[6] Ursell PG, Dhariwal M, O’Boyle D, Khan J, Venerus A (2020). 5-year incidence of YAG capsulotomy and PCO after cataract surgery with single-piece monofocal intraocular lenses: a real-world evidence study of 20,763 eyes. Eye.

[7] Nanavaty MA, Zukaite I, Salvage J (2019). Edge profile of commercially available square-edged intraocular lenses: Part 2. J Cataract Refr Surg.

[8] Hecht I, Karesvuo P, Achiron A, Elbaz U, Tuuminen R (2020). Anti-inflammatory medication after cataract surgery and posterior capsular opacification. Am J Ophthalmol.

[9] Liu S, Zhao X, Tang J, Han Y, Lin Q (2021). Drug-eluting hydrophilic coating modification of intraocular lens via facile dopamine self-polymerization for posterior capsular opacification prevention. ACS Biomater Sci Eng.

[10] Han Y, Tang J, Xia J, Wang R, Qin C, Liu S, Zhao X, Chen H, Lin Q (2019). Anti-adhesive and antiproliferative synergistic surface modification of intraocular lens for reduced posterior capsular opacification. Int J Nanomed.

[11] Gonzalez-Chomon C, Braga MEM, de Sousa HC, Concheiro A, Alvarez-Lorenzo C (2012). Antifouling foldable acrylic IOLs loaded with norfloxacin by aqueous soaking and by supercritical carbon dioxide technology. Eur J Pharm Biopharm.

[12] Topete A (2020). Dual drug delivery from hydrophobic and hydrophilic intraocular lenses: in-vitro and in-vivo studies. J Control Release.

[13] Topete A, Saramago B, Serro AP (2021). Intraocular lenses as drug delivery devices. Int J Pharm.

[14] Huang H, Zhu S, Han Y, Liu D, Liu S, Lu D, Wang R, Lin Q (2022). Cascade catalytic platform modified intraocular lens for high-efficient posterior capsule opacification prevention. Chem Eng J.

[15] Lin YX, Hu XF, Zhao Y, Gao YJ, Yang C, Qiao SL (2017). Photothermal ring integrated intraocular lens for high-efficient eye disease treatment. Adv Mater.

[16] Pang Y, Wei C, Li R, Wu Y, Liu W, Wang F, Zhang X, Wang X (2019). Photothermal conversion hydrogel based mini-eye patch for relieving dry eye with long-term use of the light-emitting screen. Int J Nanomed.

[17] Rayalu SS, Jose D, Mangrulkar PA, Joshi M, Hippargi G, Shrestha K, Klabunde K (2014). Photodeposition of AuNPs on metal oxides: study of SPR effect and photocatalytic activity. Int J Hydrogen Energ.

[18] Yang W, Liang H, Ma S, Wang D, Huang J (2019). Gold nanoparticle based photothermal therapy: development and application for effective cancer treatment. Sustain Mater Tech.

[19] Yang K, Zhao S, Li B, Wang B, Lan M, Song X (2022). Low temperature photothermal therapy: advances and perspectives. Coordin Chem Rev.

[20] Li Z, Rong L (2021). A homotypic membrane-camouflaged biomimetic nanoplatform with gold nanocrystals for synergistic photothermal/starvation/immunotherapy. ACS appl mater inter.

[21] Zhang H, Liang X, Han L, Li F (2018). "Non-Naked" gold with glucose oxidase-like activity: a nanozyme for tandem catalysis. Small.

[22] Chen J, Ma Q, Li M, Chao D, Huang L, Wu W, Fang Y, Don S (2021). Glucose-oxidase like catalytic mechanism of noble metal nanozymes. Nat Commun.

[23] Gao S, Lin H, Zhang H, Yao H, Chen Y, Shi J (2019). Nanocatalytic tumor therapy by biomimetic dual inorganic nanozyme-catalyzed cascade reaction. Adv Sci.

[24] Ploetz E, Engelke H, Lächelt U, Wuttke S (2020). The chemistry of reticular framework nanoparticles: MOF, ZIF, and COF materials. Adv Funct Mater.

[25] Wu M-X, Yang Y-W (2017). Metal-organic framework (MOF)-based drug/cargo delivery and cancer therapy. Adv Mater.

[26] Yuan S, Peng J, Cai B, Huang Z, Garcia-Esparza AT, Sokaras D, Zhang Y, Giordano L, Akkiraju K (2022). Tunable metal hydroxide–organic frameworks for catalysing oxygen evolution. Nat Mater.

[27] Nguyen HL (2022). Metal-organic frameworks can photocatalytically split water—why not?. Adv Mater.

[28] Kumar P, Anand B, Tsang YF, Kim K-H, Khullar S, Wang B (2019). Regeneration, degradation, and toxicity effect of MOFs: opportunities and challenges. Environ Res.

[29] Stockwell BR, Angeli J, Bayir H (2017). Ferroptosis: a regulated cell death nexus linking metabolism, redox biology, and disease. Cell.

[30] Chen X, Kang R, Kroemer G, Tang D (2021). Broadening horizons: the role of ferroptosis in cancer. Nat Rev Clin Oncol.

[31] Liu D, Tang J, Shen L, Liu S, Zhu S, Wen S, Lin Q (2022). Foldable bulk anti-adhesive polyacrylic intraocular lens material design and fabrication for posterior capsule opacification prevention. Biomacromol.

[32] Hong Y, Zou H, Hu Y, Fei F, Liang L, Liu D, Han Y, Lin Q (2022). Design of foldable, responsively drug-eluting polyacrylic intraocular lens bulk materials for prevention of postoperative complications. J Mater Chem B.

[33] Xiang Y, Jin R, Zhang Y, Li K, Liu G, Song X, Wang Y, Nie Y (2021). Foldable glistening-free acrylic intraocular lens biomaterials with dual-side heterogeneous surface modification for postoperative endophthalmitis and posterior capsule opacification prophylaxis. Biomacromol.

[34] Xia J, Lu D, Han Y, Wang J, Hong Y, Zhao P, Fang Q, Lin Q (2021). Facile multifunctional IOL surface modification via poly (PEGMA-co-GMA) grafting for posterior capsular opacification inhibition. RSC Adv.

[35] Han Y, Xu X, Wang Y, Liu S, Zhao X, Chen H, Lin Q (2018). Drug eluting intraocular lens surface modification for PCO prevention. Colloid Interfac Sci Commun.

[36] Liu ZJ, Li T, Han F, Gan Y, Li Y (2019). A cascade-reaction enabled synergistic cancer starvation/ROS-mediated/chemo-therapy with enzyme modified Fe-based MOF. Biomater Sci.

[37] Hu WC, Younis MR, Zhou Y, Wang C, Xia XH. Antibacterial Therapy: in situ fabrication of ultrasmall gold nanoparticles/2D MOFs hybrid as nanozyme for antibacterial therapy. Small 2020. 10.1002/smll.202000553. 10.1002/smll.202000553 32372554

[38] Ward EJ, Lacey J, Crua C, Dymond MK, Sandeman S (2020). 2D titanium carbide (Ti_3_C_2_T_x_) in accommodating intraocular lens design. Adv Funct Mater.

[39] Lischke R, Sekundo W, Wiltfang R, Bechmann M, Kreutzer TC, Priglinger SG, Dirisamer M, Luft N (2022). IOL power calculations and cataract surgery in eyes with previous small incision lenticule extraction. J Clin Med.

[40] Xie R, Weisen AR, Lee Y (2020). Glass transition temperature from the chemical structure of conjugated polymers. Nat Commun.

[41] Qie J (2022). A polydopamine-based photodynamic coating on the intraocular lens surface for safer posterior capsule opacification conquering. Biomater Sci.

[42] Tang D, Kroemer G (2020). Ferroptosis. Curr Biol.

[43] Deng X, Shao Z, Zhao Y (2021). Solutions to the drawbacks of photothermal and photodynamic cancer therapy. Adv Sci.

