# Supplementary material for: Cascade reaction triggering and photothermal AuNPs@MIL MOFs doped intraocular lens for enhanced posterior capsular opacification prevention
Source: J Nanobiotechnology. 2023 Apr 24;21:134. doi: 10.1186/s12951-023-01897-0 (PMC10127092; doi:10.1186/s12951-023-01897-0)
Supplement: Supplementary file 1 — Additional file 1: Figure S1. Particle size diagram of MIL and AuNPs@MIL measured by DLS. Figure S2. Image of the IOL made from PGE placed on an optical resolution plate (objective magnification 0.75$\times$×). Figure S3. The amperometric I-t curve of AuNPs@MIL (1 mg/mL) and H2O in presence of continuous addition of 10 mg/mL glucose solution at certain time intervals. [file 12951_2023_1897_MOESM1_ESM.docx]

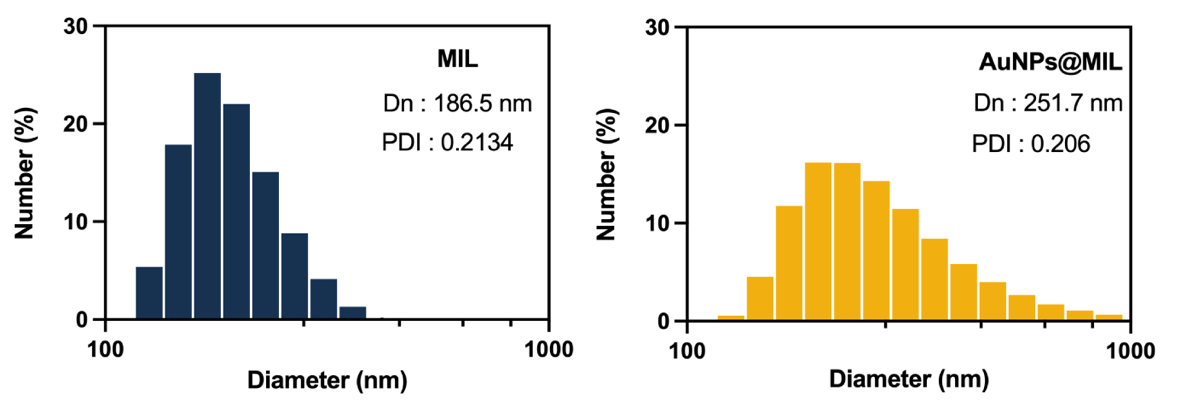


Figure S1. Particle size diagram of MIL and AuNPs@MIL measured by DLS.


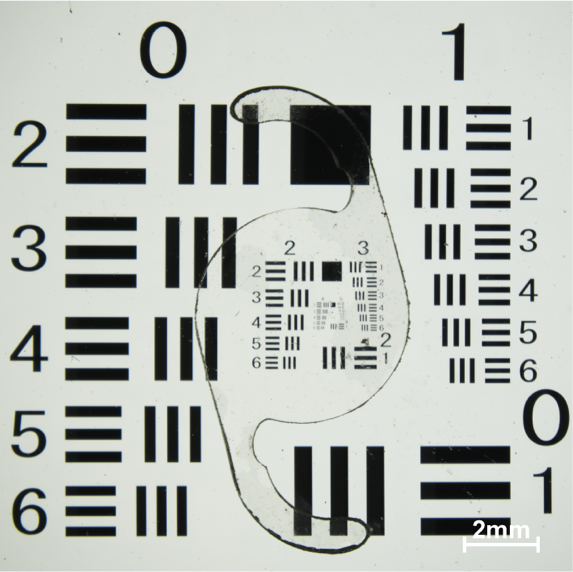


Figure S2. Image of the IOL made from PGE placed on an optical resolution plate (objective magnification 0.75$\times$) .


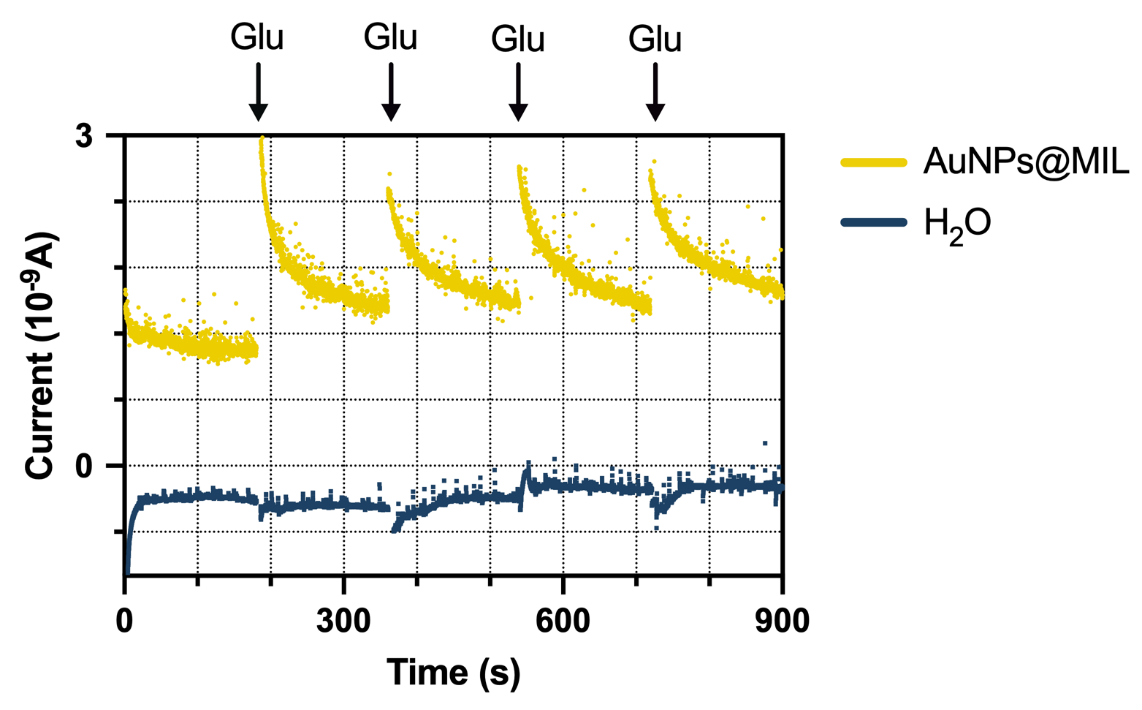


Figure S3. The amperometric I-t curve of AuNPs@MIL (1 mg/mL) and H_2_O in presence of continuous addition of 10 mg/mL glucose solution at certain time intervals.
